# Neoplastic transformation of rat liver epithelial cells is enhanced by non-transferrin-bound iron

**DOI:** 10.1186/1471-230X-8-2

**Published:** 2008-02-06

**Authors:** Donald J Messner, Kris V Kowdley

**Affiliations:** 1 Bastyr University, 14500 Juanita Drive, Kenmore, WA 98028, USA; 2 Benaroya Research Institute and Virginia Mason Medical Center, Seattle, WA 98101, USA

## Abstract

**Background:**

Iron overload is associated with liver toxicity, cirrhosis, and hepatocellular carcinoma in humans. While most iron circulates in blood as transferrin-bound iron, non-transferrin-bound iron (NTBI) also becomes elevated and contributes to toxicity in the setting of iron overload. The mechanism for iron-related carcinogenesis is not well understood, in part due to a shortage of suitable experimental models. The primary aim of this study was to investigate NTBI-related hepatic carcinogenesis using T51B rat liver epithelial cells, a non-neoplastic cell line previously developed for carcinogenicity and tumor promotion studies.

**Methods:**

T51B cells were loaded with iron by repeated addition of ferric ammonium citrate (FAC) to the culture medium. Iron internalization was documented by chemical assay, ferritin induction, and loss of calcein fluorescence. Proliferative effects were determined by cell count, toxicity was determined by MTT assay, and neoplastic transformation was assessed by measuring colony formation in soft agar. Cyclin levels were measured by western blot.

**Results:**

T51B cells readily internalized NTBI given as FAC. Within 1 week of treatment at 200 μM, there were significant but well-tolerated toxic effects including a decrease in cell proliferation (30% decrease, p < 0.01). FAC alone induced little or no colony formation in soft agar. In contrast, FAC addition to cells previously initiated with N-methyl-N'-nitro-N-nitrosoguanidine (MNNG) resulted in a concentration dependent increase in colony formation. This was first detected at 12 weeks of FAC treatment and increased at longer times. At 16 weeks, colony formation increased more than 10 fold in cells treated with 200 μM FAC (p < 0.001). The iron chelator desferoxamine reduced both iron uptake and colony formation. Cells cultured with 200 μM FAC showed decreased cyclin D1, decreased cyclin A, and increased cyclin B1.

**Conclusion:**

These results establish NTBI as a tumor promoter in T51B rat liver epithelial cells. Changes in cyclin proteins suggest cell cycle disregulation contributes to tumor promotion by NTBI in this liver cell model.

## Background

Iron is an essential metal, but is potentially toxic and therefore tightly regulated in mammalian systems [1,2]. Most body iron stores are sequestered in a non-toxic form through high affinity binding to transport and storage proteins including transferrin and ferritin. There is also a significant pool of "free iron" complexed to low molecular weight (MW) carriers such as citrate. Intracellular free iron is a necessary intermediate between iron storage depots and biosynthetic pathways that utilize iron. It also mediates translational control of iron homeostasis by binding to iron regulatory proteins. However, free iron can undergo redox cycling, forming reactive oxygen species (ROS) through the Fenton and Haber Weiss reactions [3-5]. ROS damage biomolecules and cause oxidative stress by depleting cellular antioxidant stores and may result in cell death [6-8]. The potential for iron damage is particularly high in liver, the primary organ for storage of excess iron [2]. As there is no significant excretion of iron, excess uptake may be accompanied by severe liver damage that progresses to liver failure or hepatocellular carcinoma (HCC) [9]. This occurs in diseases of iron overload, including hereditary hemochromatosis. Elevated liver iron is also associated with increased HCC in other liver diseases, including biliary cirrhosis and hepatitis C [10].

Iron overload is marked by increases in both transferrin-bound and free, non-transferrin-bound iron (NTBI) in blood [11,12]. Several considerations suggest these two forms are separable: (1) Although transferrin-bound iron has significant growth-promoting effects, stimulation of cell growth by NTBI was seen only over a narrow concentration range and under transferrin-limiting conditions [13,14]. Tumor cells, which can have increased growth rates, frequently have increased levels of transferrin receptors [15], and iron uptake via this route is higher than in neighboring cells. Yet in iron overload, tumors contain lower iron levels than surrounding liver tissue [16]. Similarly bone marrow cells, with a higher level of transferrin receptors than hepatocytes, do not accumulate iron in iron overload diseases. (2) Humans and animals that lack transferrin still develop iron overload [17]. (3) There are many reports of experimental iron overload in cells and animals given NTBI [6-8], but not transferrin-bound iron. (4) Rodent studies identified transferrin-independent pathways of iron uptake in liver [18,19]. This was confirmed in rat hepatocytes and other mammalian cell types in culture [20-23]. (5) Finally, unlike transferrin uptake via receptor-mediated endocytosis, NTBI uptake was not downregulated in iron replete cells; it increased with exposure to iron [24-26]. In addition, NTBI uptake in liver increased in an animal model of hemochromatosis [27]. These points suggest that mechanisms that don't involve transferrin receptors are critical for iron overload in liver. NTBI is an important, and possibly the primary, source of iron-related toxicity in liver.

Although transferrin-independent uptake and toxicity of NTBI have been demonstrated in animals and in cultured cells, effects on neoplastic transformation are not understood. This is due partly to a lack of suitable experimental models, and partly to the difficulty of obtaining effects using physiological forms of NTBI. The high rate of HCC among human hemochromatosis patients with cirrhosis has not been replicated in animal models of this disease [2,9]. Non-physiological forms of dietary iron contributed to liver cancer in animals [28,29], but the relevance to biological iron is unknown. Similarly, prior reports that iron acts as a co-carcinogen or tumor promoter in liver and cultured cells also depended on non-physiological iron ligands [16,30,31]. In some protocols iron inhibited or had no effect on cell transformation [32,33]. Transformation protocols that require any form of iron have not been established in human cells. No previous studies have reported transformation-related effects of iron administered in a form that is present in humans.

Ferric citrate is present in blood and its levels increase in hereditary hemochromatosis [12,34]. It may be an important contributor to the pathological effects of iron overload in humans, including hepatocellular carcinoma. Ferric ammonium citrate (FAC) is a formulation that minimizes generation of insoluble iron hydroxides *in vitro *[35]. The present study investigated the transforming effects of this physiologically and pathologically relevant form of NTBI. We utilized T51B rat liver epithelial cells, a well-characterized model for tumor promotion and carcinogenicity studies. We found FAC has properties of a tumor promoter, rather than a complete carcinogen. Iron-induced changes in cyclin proteins suggest tumor promotion results in part from disruptions in regulation of the T51B cell cycle in proliferating cells.

## Methods

### Materials

N-methyl-N'-nitro-N-nitrosoguanidine (MNNG), ferric ammonium citrate (FAC), desferoxamine, and calcein-AM were from Sigma/Aldrich (St. Louis, MO). Newborn calf serum was from Atlanta Biologicals (Norcross GA). Other cell culture reagents were from GIBCO/Invitrogen (Carlsbad, CA). Agarose was from Cambrex BioScience (Rockland, ME). Antibodies and other specialty reagents were from commercial sources as noted below. Concentrated stock solutions were prepared assuming 100% reagent purity and stored in aliquots at -20°C. Solutions in solvent were kept at -20°C until use, while aqueous reagents were used after thawing and storage at 4°C for limited periods. As appropriate, control experiments were run to document that solvent alone had no effect. MNNG stock solutions were freshly prepared just prior to use.

### Cell culture and transformation assays

T51B cells are a non-neoplastic cell line derived from rat liver and used in studies of carcinogenicity and tumor promotion [36-39]. T51B cells were maintained in Eagles basal media supplemented with 10% newborn calf serum, 2 mM l-glutamine, and 100 U/ml penicillin/streptomycin (complete media), at 37°C in a 5% CO2 atmosphere. For proliferation and all other assays, treatment was started 1 day after plating. In general, untreated cells reached confluence 5 days after plating and were subsequently quiescent [38]. Cell number was determined at 3 days treatment (to approximate log phase growth rate) and at 7 days treatment (to approximate saturation density) by trypsinization and counting with a hemocytometer. Control experiments demonstrated >95% of the cells were viable as determined by trypan blue exclusion. Multiple replicates were compiled for statistical analysis and presentation. To determine differences between untreated and FAC-treated groups, the data were evaluated using a 2 tailed unpaired student t-test for samples with unequal variance, and significance noted at p < 0.01 and p < 0.001 levels.

Toxicity assays used the MTT method [40] in a 96 well plate format at an initial seeding density of 10,000 cells per well. Treatments were initiated 1 day after plating and renewed in fresh complete media after 2 days. After treatment for 5 days, cells were rinsed with PBS and incubated with 0.3 μg/ml methylthiazolyldiphenyl-tetrazolium bromide (MTT) in complete media containing 10 mM HEPES pH7.4 for 3 hours. The formazan product was solubilized in DMSO and measured by absorbance at 540 nm. Statistical evaluations (to compare treated to untreated cells cultured in parallel) were performed as described above for cell proliferation.

For the transformation assays, the cells were treated with or without 0.5 μg/ml MNNG one day after plating. After 24 hours, the media was renewed and test treatments initiated. Cells receiving MNNG only were cultured in complete media for the same times as cells receiving test tumor promotion treatments. The cells were passaged every 2 weeks during the transformation experiments, and the media/treatments were further renewed 3–4 times between each splitting. Starting at 12 weeks, aliquots of cells were plated in soft agar to assess transformation [38]. Specifically, colony formation in soft agar was measured after 12, 14, and 16 weeks in monolayer culture (experiment 1); after 12, 14, 16, 18, and 20 weeks (experiment 2); and after 12, 14, 16, and 18 weeks (experiments 3 and 4). An aliquot corresponding to 25,000 cells prepared in 0.35% top agar media (agarose in Iscove's DMEM containing 10% newborn calf serum and 10 ng/ml epidermal growth factor) was layered on 0.6% bottom agar media in a 60 mm dish. After 3 weeks the colonies were stained with 1 ml 0.5 mg/ml iodonitrotetrazolium violet and counted under the microscope. Colonies larger than 0.17 mm in diameter (approximately 100 cells) were scored as positive. The means and standard error of the means (s.e.m.) were determined from quadruplicate soft agar plates in single experiments or after compiling data from multiple experiments as specified. For clarity, data from selected but representative time points are presented for some experiments. Statistical evaluations (to compare experimental to control cells cultured in parallel) were performed as described above for cell proliferation.

### Biochemical measurements

Total non-heme iron content of cells was determined using ferrozine [41] as follows. Cells were rinsed in PBS on ice, and then lysed and scraped in deionized water. One volume of buffer A (1 M HCl 10%TCA) was added and the sample was heated at 95°C for 30–45 minutes. Samples were cooled to room temperature and the protein precipitate removed by centrifugation for 10 minutes at 14,000 × g. An aliquot of the supernatant was mixed with 1 volume of buffer B (0.58 mM ferrozine, 1.5% thioglycolic acid, 1.5 M sodium acetate) and incubated for 30 minutes at room temperature. The absorbance of samples at 570 nm was compared in duplicate to an NIST-traceable iron reference (Fluka) standard curve run in parallel. Approximately 0.75 nmol iron was required to achieve less than 10% deviation from the standard curve, corresponding to a limit of detection of roughly 2 nmol/mg protein. The non-heme iron content of untreated control cells was below this limit and must be considered an approximation. Qualitatively similar results, but with less sensitivity, were obtained using the bathophenanthroline disulfonate assay [26]. Cell lysate protein was determined relative to BSA by a modified Lowry assay. Procedures for western blot analysis, including cell harvesting, have been described [38,42]. Antibodies to ferritin heavy and light chains, cyclin B1, cyclin D1, cyclin E, and GAPDH, were from Santa Cruz Biotechnology (Santa Cruz, CA). Antibodies to cyclin A were from Oncogene Research Products (Cambridge, MA). Secondary antibodies linked to horseradish peroxidase were from Jackson Immunoresearch (West Grove, PA). Detection utilized the ECL-plus system from Amersham/GE Healthcare (Arlington Heights, IL).

Calcein fluorescence in cells was assessed by epifluorescence microscopy. Cells plated on glass coverslips were treated for 30 minutes with 0.25 μg/ml calcein-AM (Sigma) in serum-free media, rinsed, and incubated in complete media with or without FAC and dfo for the times indicated. The fluorescence of intracellular calcein is quenched by the influx of free iron. Although calcein may also be partially degraded by iron under certain conditions [43], this would minimally require iron uptake by the cells. Loss of calcein fluorescence by either mechanism (quenching or degradation) indicates iron influx. The coverslips were rinsed 3× in PBS, mounted using Vectashield (Vector Laboratories, Burlingame, CA), and viewed with a Nikon Eclipse 50i inverted microscope equipped with X-cite 120 epifluorescence. For each treatment condition, identical fields were photographed with a Nikon Coolpix 4500 digital camera to record FITC fluorescence and phase contrast views. Constant photographic parameters (exposure, contrast, magnification, etc.) were maintained for all treatment conditions.

## Results

### Non-transferrin bound iron uptake in T51B cells

**Figure 1:**
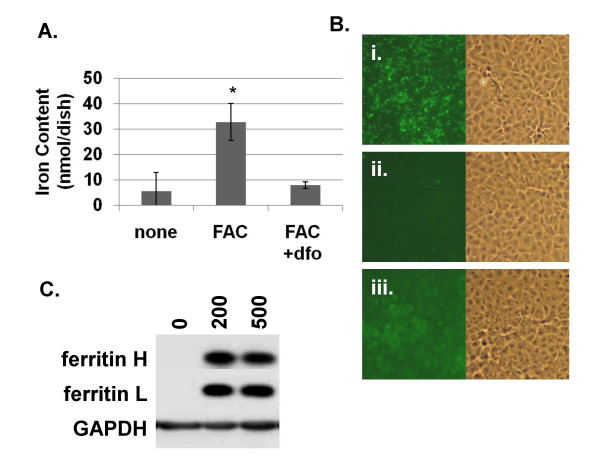
**Characterization of non-transferrin-bound iron internalization in T51B liver epithelial cells**. **A**. **Non-heme iron content**. Cells were left untreated (none) or treated with 200 μM ferric ammonium citrate (FAC) or with 200 μM FAC and 160 μM desferoxamine (FAC + dfo) for 5 days. Total non-heme iron (nmol/mg cell lysate protein) was determined by a ferrozine-based colorimetric assay as described under Methods. Values are reported as the means +/- s.e.m. of triplicate dishes (**p < 0.001 compared to control). **B**. **Quenching of calcein fluorescence**. Cells were pulsed with calcein-AM for 30 minutes, rinsed, and incubated for 2 days in complete cell media containing: **(i) **no addition, **(ii) **200 μM FAC, or **(iii) **200 μM FAC and 160 μM dfo. Identical fields for FITC fluorescence (left panels; corresponding to calcein signal) and phase contrast (right panels) are shown. **C**. **Ferritin content**. Cells were treated with 0, 200, or 500 μM FAC for 5 days and processed for western blots using antibodies specific for ferritin L, ferritin H, or GAPDH as gel loading control. Each experiment was performed at least twice with similar results.

T51B is a non-neoplastic liver epithelial cell line used for transformation and tumor promotion studies [37,38,44]. This cell line was derived from adult rat liver and is similar in morphology and marker protein content to liver oval epithelial cells [36,39,45]. Uptake of NTBI has not been previously demonstrated in liver oval cells. Iron uptake in T51B cells was characterized by three approaches as illustrated in Figure 1. First, a chemical assay of non-heme iron content showed a dramatic increase in cells treated with FAC for 5 days (Figure 1A) that was reduced by the specific iron chelator desferoxamine (dfo). Second, the fluorescence signal from the iron-sensitive dye calcein was lost upon incubation of the cells with FAC (Figure 1B). This occurred within 48 hours of FAC addition and was also inhibited by dfo. Finally, western blotting showed both ferritin H and ferritin L increased in FAC-treated cells (Figure 1C). The ferritin increase occurred within 48 hours of addition of 200 μM FAC, was dfo-sensitive, and was maintained for at least 12 weeks of culture in FAC, the longest time point examined (not shown). These experiments demonstrated that iron given as FAC readily accumulates in T51B cells in a uniform fashion with expected effects on cellular pathways regulating iron metabolism.

**Figure 2:**
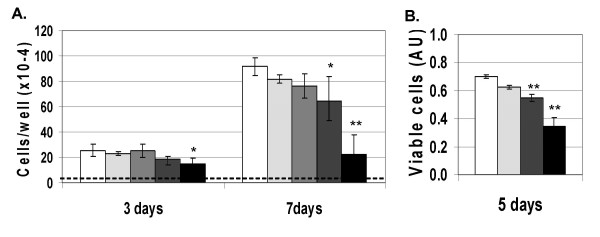
**Anti-proliferative effects of FAC**. T51B cells were treated with FAC in complete culture media starting one day after plating. The FAC concentrations were: none (open bars), 50 μM (light stipled bars), 100 μM (medium stipled bars), 200 μM (dark stipled bars), and 500 μM (solid bars). **A**. **Cell number**. Triplicate wells were harvested and counted after 3 days or after 7 days (with one renewal of FAC in fresh media). The dashed line indicates the plating density of 40,000 cells per well. **B**. **Cell viability**. Triplicate wells were assayed using MTT after 5 days as described under Methods. In all panels the means and standard errors determined from three separate experiments are shown (*p<0.01, **p<0.001 compared to untreated control cells).

The proliferation rates of subconfluent T51B cells were examined to identify a concentration range of FAC suitable for carcinogenesis and tumor promotion studies. Figure 2A shows there was little effect over the first three days of treatment at the concentrations examined. However, by 7 days a significant dose-related growth inhibition was apparent. Nearly complete growth arrest was seen at 500 μM FAC (Figure 2, compare number of cells at 3 and 7 days). In contrast, 200 μM FAC appeared well-tolerated by T51B cells for extended periods. After 7 days at this concentration, cell proliferation was significantly slowed compared to untreated cells (30% decrease, p < 0.01) but was not blocked. Similar dose-dependent effects of FAC were seen when cell toxicity was measured by MTT assay, which reflects cell viability as well as number (Figure 2B). The modest effect at 200 μM (a 22% decrease at 5 days) was much greater at 500 μM FAC (51% decrease; p < 0.001). At this concentration FAC was unacceptably toxic to the cells. These experiments defined an upper limit for the transformation experiments: the dose of FAC (given with each media renewal) at which T51B cells can be subcultured and continue to grow.

### FAC acts as a tumor promoter in T51B cells

**Figure 3:**
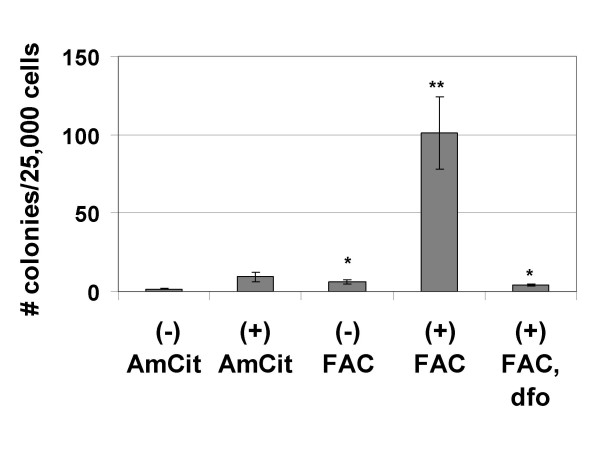
**Iron acts as a tumor promoter in T51B cells**. Cells treated with (+) or without (-) 0.5 μg/ml MNNG as initiating agent were promoted for an additional 16 weeks in four separate experiments. Media additions during the promotion phase and the number of replicates were: 200 μM ammonium citrate (AmCit), n = 4 (- MNNG), n = 4 (+ MNNG); 200 μM ferric ammonium citrate (FAC), n = 3 (- MNNG), n = 4 (+ MNNG); or 200 μM ferric ammonium citrate,160 μM desferoxamine (FAC, dfo), n = 2 (+ MNNG). Cell transformation was assayed by growth in soft agar as described under Methods. Mean values and standard errors from up to four separate experiments are shown (*p<0.01, **p<0.001 compared to AmCit only controls).

Growth in soft agar is an *in vitro *indicator of neoplastic transformation [46]. If iron is a complete carcinogen in T51B cells, then FAC treatment alone should cause these non-neoplastic cells to form colonies in soft agar. If iron is a tumor promoter, then prior initiation of the cells is required. To test for these possibilities, we examined soft agar growth of FAC-treated cells with and without initiation by N-methyl-N'-nitro-N-nitrosoguanidine (MNNG). The effects of MNNG at different FAC concentrations are shown in Table 1. Colony formation was greatest in MNNG-initiated cells also promoted with FAC. There was a dose-dependent effect of FAC: colony formation increased from 20 μM to 200 μM FAC. Further increase to the more toxic concentration of 500 μM FAC was less effective. Table 2 shows results from a second experiment, evaluating the time dependence of promotion at two FAC concentrations. As seen in experiment 1, colony formation at 12 weeks was greatest in cells exposed to MNNG and 200 μM FAC. This increased with time of promotion (Table 2, compare 200 μM FAC values from 12 to 20 weeks). Time dependence was also seen at 50 μM FAC, though it was lesser in magnitude and delayed. Cells treated with MNNG and 200 μM ammonium citrate (i.e. without iron) showed no significant colony formation (Table 2). Altogether, results from four independent experiments demonstrated that optimal colony formation required initiation by MNNG and promotion by FAC (Figure 3). It was much less apparent if iron was omitted from the protocol or chelated by desferoxamine. These data indicate that NTBI administered as FAC is a tumor promoter, but not a complete carcinogen, in T51B cells.

**Table 1:** Transformation of T51B cells by MNNG and FAC^1^.

**MNNG**	**FAC**	**Number of colonies per 25,000 cells**
0	0	0.0 (+/- 0.0)
0.5	0	1.3 (+/- 0.8)
0	20 μM	1.5 (+/- 0.5)
0.5	20 μM	4.3 (+/- 1.0)
0	50 μM	1.8* (+/- 0.2)
0.5	50 μM	17.5 (+/- 3.3)
0	200 μM	1.8 (+/- 1.4)
0.5	200 μM	21.5* (+/- 2.1)
0	500 μM	3.0 (+/- 1.4)
0.5	500 μM	5.3 (+/- 1.3)

^1 ^Cells exposed or not to 0.5 μg/ml MNNG were cultured in FAC for 12 weeks (6 passages) prior to soft agar assay: means of n = 4 dishes (+/- s.e.m); *p < 0.01 compared to untreated cells.

**Table 2:** Time dependence of tumor promotion by iron.

**MNNG**	**Promoter**	**Number of soft agar colonies per 25,000 cells**^1^				
		**Week 12**	**Week 14**	**Week 16**	**Week 18**	**Week 20**
0	50 μM FAC	0.8 (+/- 0.5)	0.8 (+/- 0.5)	3.5 (+/- 1.2)	2.3 (+/- 0.5)	4.0 (+/- 2.0)
0.5	50 μM FAC	7.5 (+/- 1.4)	4.5*(+/- 0.5)	7.0*(+/- 0.4)	17.8*(+/- 1.3)	52.7*(+/- 1.2)
0	200 μM FAC	5.3 (+/- 1.2)	1.0 (+/- 0.4)	10.8 (+/- 2.4)	9.3 (+/- 1.0)	2.3 (+/- 1.2)
0.5	200 μM FAC	12.8*(+/- 1.0)	21.5*(+/- 0.3)	32.5 (+/- 8.5)	66.0*(+/- 9.0)	>99* (n.d.)
0	200 μM AmCit	1.5 (+/- 0.3)	0.0 (+/- 0.0)	1.3 (+/- 0.6)	0.0 (+/- 0.0)	0.3 (+/- 0.3)
0.5	200 μM AmCit	1.3 (+/- 0.5)	0.0 (+/- 0.0)	0.5 (+/- 0.3)	0.0 (+/- 0.0)	0.7 (+/- 0.7)

^1^Cells exposed or not to 0.5 μg/ml MNNG were cultured with FAC (or Ammonium Citrate control) for the indicated times, with 2 week passaging intervals, prior to soft agar assay (means of n = 4 soft agar dishes; *p < 0.01 compared to 200 μM AmCit only controls). The agar dishes from cells treated with 0.5 μg/ml MNNG + 20 weeks 200 μM FAC had too many colonies to accurately count (the s.e.m. was not determined).

### Iron loading elicits changes in cell cycle proteins in T51B cells

**Figure 4:**
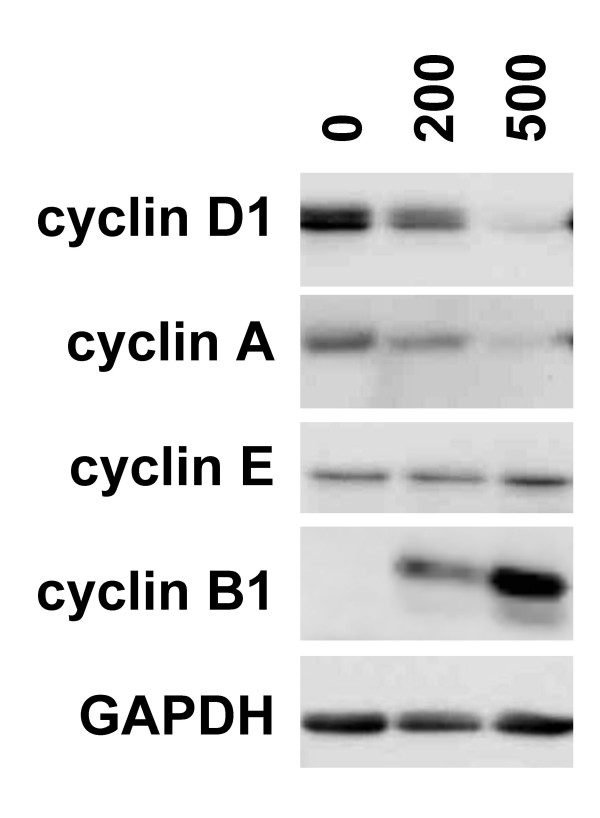
**Iron alters the cell cycle distribution of T51B cells**. Proliferating cells were treated with the indicated concentrations of FAC (in μM) for 5 days and processed for western blots using antibodies specific for cyclin D1, cyclin E, cyclin A, cyclin B1, and GAPDH as gel loading control. All panels are from a single experiment and are representative of results obtained at least 4 times.

At tumor promoting concentrations, FAC did not increase T51B cell proliferation, but rather inhibited it slightly (Figure 2A). The levels of cyclin proteins should inform on the nature of this effect and may suggest a basis for tumor promotion [47]. Figure 4 shows the effect of FAC on the levels of cyclin proteins in T51B cells. Modest reductions in a G1-phase cyclin (D1) and the S-phase cyclin (A) correlated well with the slight decrease in proliferation seen in cells exposed to tumor promoting concentrations of FAC (200 μM). And, a more complete loss from cells exposed to 500 μM FAC correlated well with the proliferative block seen at that concentration. There was a concomitant decrease in junB levels and AP-1 activity (data not shown) that may partially explain these decreases. Other cyclins not primarily regulated by AP-1, including a G1 cyclin (E) and the M-phase cyclin (B1), did not decrease in FAC-treated cells. Cyclin E levels remained unchanged, while cyclin B1 levels increased significantly (Figure 4). The increase in cyclin B1 was not seen in cells treated with ammonium citrate controls or when the bulk of the iron was chelated by dfo (not shown).

## Discussion

The current study demonstrated that FAC acts as a tumor promoter in T51B liver cells. We also found tumor-promoting concentrations of FAC decreased, rather than increased, the proliferation of normal T51B cells. To our knowledge, this is the first report to describe tumor promotion by a physiologically and pathologically relevant form of iron. This is an important point missing from earlier studies of iron overload and neoplastic transformation. Previous experiments used various non-physiological chelating ligands to increase the bioavailability of iron. For example, carbonyl iron caused moderate iron overload in rats, but ferrocene was required for severe iron overload, liver neoplasms, and HCC [28,29]. Iron given as the nitrilotriacetate (NTA) complex caused DNA damage and transformation of cells in culture, whereas iron citrate did not [31,48,49]. Iron-NTA was a liver tumor promoter in rats [30]. Co-administration of an iron ionophore significantly increased iron effects [35]. Until now it was unclear whether ionic iron alone had transformation-related effects in a mammalian system. Our finding, that FAC had tumor promotion activity in the absence of non-physiological chelating ligands, settles this dispute. This has potential clinical implications, as the goal to reduce the incidence of HCC among iron overload patients may be accomplished through long-term reduction of iron levels [9]. Novel strategies that target NTBI may be particularly effective in achieving this goal.

By definition, tumor promotion involves the selective proliferation of pre-neoplastic (vs. normal) cells. Classical tumor promoters such as phorbol 12-myristate 13-acetate (TPA) increase DNA synthesis and cell proliferation in cell and animal models of carcinogenesis [50]. This mitogenic effect is thought to be critical for tumor promotion, acting by positive selection to increase proliferation of initiated cells. Cell proliferation is needed to fix and clonally expand carcinogenic mutations resulting from chemically-induced DNA damage. Alternatively, a tumor promoter may cause growth inhibition and/or cell toxicity, accompanied by outgrowth of a resistant phenotype. This idea was first proposed for liver by Farber and co-workers [51,52] as the "resistant hepatocyte model" of tumor promotion. Similarly, a role for compensatory proliferation in liver tumor promotion has been proposed [53,54]. Essentially, a certain degree of cell toxicity is tumor promoting in liver because it allows for compensatory proliferation of chemically initiated cells, which would otherwise remain quiescent. These previously described negative selection models are consistent with our findings and offer insight into how NTBI may contribute to HCC in iron overload. We propose that anti-proliferative or other toxic effects of iron loading on normal cells, rather than mitogenic effects on pre-neoplastic cells, explain tumor promotion in the T51B cell model. Consequently, agents which prevent NTBI toxicity are predicted to also block tumor promotion.

HCC may originate from hepatocytes or oval cells, a precursor stem cell type in liver [55-61]. Differentiated hepatocytes do not readily proliferate in culture, and so are not suitable for the type of study presented here. To model iron-related HCC, therefore, we used T51B cells, a cell type similar to liver oval cells. In addition, we used 50–200 μM FAC for 12–16 weeks to establish iron overload. Although development of HCC in humans with hemochromatosis occurs at lower serum iron citrate concentrations (5–20 μM) over several decades [9,11,12,62,63], several considerations suggest our experimental conditions are appropriate. First, studies of serum NTBI in humans are only partially informative. Iron citrate (unlike transferrin iron) is very rapidly cleared from the blood by the liver [18], and so the serum concentration likely underestimates liver exposure. Second, iron-related HCC occurs primarily in the setting of liver cirrhosis. The effect of cirrhosis on iron citrate concentrations in the liver itself is unknown, but exposure of preneoplastic cells to levels higher than reported in blood seems possible. Finally, studies of high concentrations of carcinogens and tumor promoters given for short times are generally accepted as useful predictors of effects caused by exposure to lower concentrations for longer times. These points argue that findings from the T51B cell model are applicable to the promotion phase of iron-related HCC in humans.

The route of NTBI uptake in T51B cells is unknown, but there are several possibilities. The divalent metal transporter DMT-1 (NRAMP2) is thought to be important in most cell types [2,64-66]. This protein has been localized to the cell surface in hepatocytes [67], and iron transport at pH 7.4 has been documented [65]. However, iron transport by DMT-1 is optimal near pH 5.5, consistent with a primary function in recovery of iron released from transferrin in endosomes. In AML12 hepatocytes, the cell surface zinc transporter zip14 is an additional pathway [68]. This protein is particularly interesting with respect to neoplastic transformation, as zip14 was reported to be under expressed in HCC [69]. Downregulation of NTBI uptake is one potential mechanism by which initiated cells could minimize iron-related toxicity and gain a proliferative advantage over normal cells in our model. Alternate NTBI uptake pathways identified in other cell types include the TRP family of cell surface non-selective cation channels [70], and L-type calcium channels [71].

At present, we surmise that NTBI toxicity impaired progression of T51B cells into or through mitosis, based on high levels of cyclin B1. ROS generated by a Fenton-type reaction involving vanadate was shown previously to cause increased cyclin B and M-phase arrest [72,73]. Decreases in cyclins D1 and A are expected if proliferating cells become delayed at this point in the cycle. Importantly, these changes were evident at tumor promoting concentrations of FAC (200 μM). Relatively minor phenotypic distinctions may allow pre-neoplastic initiated cells to evade the selective pressure exerted by FAC at this concentration. However, these distinctions were insufficient to overcome additional toxic effects of higher concentrations, since tumor promotion decreased at 500 μM FAC. The cause(s) of increased cyclin B and cell cycle delay are unknown; dissecting potential mechanisms is a goal of future experiments. The step taken here, of demonstrating that these changes are caused by a physiologically and pathologically relevant form of NTBI under conditions of tumor promotion, is a critical one towards understanding and preventing iron-related carcinogenesis in humans.

## Conclusion

We conclude that NTBI is a tumor promoter, but not a complete carcinogen, in T51B rat liver epithelial cells. This study is the first demonstration that a form of iron present in humans has cancer-related effects. It defines an experimental model for future studies on mechanism and prevention of iron-related liver cancer.

## Competing interests

The author(s) declare that they have no competing interests.

## Authors' contributions

DJM helped to conceive and design the study, performed the experimental work, interpreted the results and prepared the manuscript. KVK helped to conceive the study, interpret results, and prepare the manuscript. Both authors read and approved the final manuscript.

## Pre-publication history

The pre-publication history for this paper can be accessed here:


